# Short-term low-carbohydrate diet decreases body weight and fat mass but not muscle strength in children and young people with type 1 diabetes

**DOI:** 10.1038/s41430-025-01658-2

**Published:** 2025-08-22

**Authors:** V. Neuman, K. Maratova, L. Plachy, L. Drnkova, S. Pruhova, S. Kolouskova, B. Obermannova, S. A. Amaratunga, M. Kulich, J. Havlik, O. Cinek, Z. Sumnik

**Affiliations:** 1 Department of Pediatrics, 2nd Faculty of Medicine, Charles University and Motol University Hospital, Prague, Czechia; 2 Department of Probability and Mathematical Statistics, Faculty of Mathematics and Physics, Charles University in Prague, Prague, Czechia; 3 Department of Food Science, Czech University of Life Sciences, Prague, Czechia; 4 Department of Microbiology, 2nd Faculty of Medicine, Charles University and Motol University Hospital, Prague, Czechia

**Keywords:** Metabolic disorders, Type 1 diabetes

## Abstract

**Aims:**

We investigated whether a short period of tightly controlled low-carbohydrate diet (LCD) leads to a change in body weight, body composition, and muscle strength in children and young people with diabetes (CYPwD).

**Methods:**

Thirty-five CYPwD were recruited into this randomized controlled cross-over study (20 female; age 14.5 ± 2.9 years). The interventions were five and five weeks of ready-made food box deliveries of isocaloric diets in random order: either LCD (94.5 ± 4.7 g/day) or recommended carbohydrate diet (RCD) (191 ± 19.2 g/day). The outcomes were body weight and body mass index (BMI) standard deviation scores (SDS), body fat percentage assessed by bioimpedance and muscle strength assessed by jumping mechanography at the end of each dietary intervention. The Welch two-sample t-tests were used to determine the difference in outcomes.

**Results:**

At the end of the LCD period, the participants had significantly lower body weight and BMI SDS than at the end of the RCD period (61.7 kg vs. 62.6 kg, *P* < 0.001, and 22.3 kg/m^2^ vs. 22.7 kg/m^2^, *P* < 0.001) and (0.84 SD vs. 0.94 SD, *P* < 0.001, and 0.81 SD vs. 0.91 SD, *P* < 0.001). The body fat percentage was lower at the end of the LCD period (24.5% vs. 25.3%, *P* = 0.001). Dynamic muscle functions did not differ significantly at the end of the intervention periods.

**Conclusions:**

We demonstrated that a short-term low-carbohydrate diet is able to decrease body weight, BMI, and decrease the percentage of body fat in CYPwD without negatively affecting their muscle function.

## Introduction

Type 1 diabetes (T1D) is an autoimmune disorder that requires lifelong application of insulin to maintain stable blood glucose levels. With the advent of new technologies including continuous glucose monitoring and, more recently, automated insulin delivery systems, more children and young people with T1D (CYPwD) are able to achieve metabolic targets [1]. Yet, there is still a significant gap in the life expectancy of people with or without T1D partly caused by high cardiovascular mortality [2]. As overweight and obesity are known contributors to cardiovascular risk, and their rates in pediatric population, including CYPwD are increasing [3, 4], it is crucial to identify effective strategies to counteract this concerning trend.

Low-carbohydrate diet (LCD) in children is characterized as the reduction of carbohydrate intake below 26% of daily energy intake [5]. Due to the non-physiological distribution of nutrient intake and possible associated risks including blunted response to glucagon possibly increasing the risk for severe hypoglycemia [6], disturbance of blood lipid profile [7], increased risk of eating behavior disorders [8, 9], fatigue [7] or decreasing growth velocity [7] [10], it is currently not recommended for CYPwD by the International Society for Pediatric and Adolescent Diabetes (ISPAD) which recommends 40-50% of the energy intake to come from carbohydrates [11]. These undesirable effects were often seen in very low-carbohydrate or ketogenic diet (i.e. below 10% of daily energy intake from carbohydrates) [5] but do not seem to be so pronounced with less stringent carbohydrate restriction [12]. There is also some evidence that LCD is also associated with some benefits mainly including improved glucose control [12–14] or increased quality of life [15, 16]. Due to the increasing rates of pediatric overweight and obesity, another possible benefit of LCD comes to the front – its capacity for weight reduction and the decrease of body fat which was seen in adults [17, 18]. In addition, since some of the possible undesired effects of LCD are rather subjective (fatigue), there is so far an unmet need to objectify these. Furthermore, despite the lacking evidence to support our decision about LCD, it is gaining popularity among people with T1D in hopes of improving the metabolic outcomes. [13, 19]

To assess the complex role of LCD on various aspects of health of CYPwD (including glucose control, quality of life, and tolerability) we designed a randomized controlled trial published previously [12]. The current study is a sub-analysis primarily focused on anthropometric features.

The aim of this paper is to assess the changes in body weight, BMI, body composition and muscle strength in a setting of a randomized controlled cross-over trial with tightly controlled LCD in CYPwD.

## Methods

### Study design and population

Thirty-five children and young people with type 1 diabetes (20 female; median age 14.5 ± 2.9 years) were prospectively recruited into this randomized controlled trial with crossover design. The details on the study population and study design are described elsewhere [12]. Briefly, the participants underwent 5 weeks of low-carbohydrate diet (LCD, i.e. 15% of their age and sex specific recommended energy intake from carbohydrates) and 5 weeks of recommended carbohydrate diet (RCD, i.e. 35-45% of children’s age and sex specific recommended energy intake from carbohydrates) in with the order of the diets randomly allotted by each participant. The energy intake for each participant was calculated based on sex and age specific dietary recommendations [11] and the subjects were pooled into four categories, 6300 kJ, 7100 kJ, 7900 kJ and 8700 kJ. Despite the different amount of carbohydrates, the diets were designed to be isocaloric for both intervention periods. Five meals per day were prepared by an experienced ready-made diet company (Nutric Bistro, Prague, Czechia) and delivered in pre-made boxes to the participants’ homes for both of the intervention periods. The participants were advised to finish their meals and not add additional carbohydrates except as rescue carbohydrates for hypoglycemia. The participants kept written information on decreased or added carbohydrates which were used for the macronutrients analysis together with detailed information from the dietary company.

The study protocol was registered at ClinicalTrials.gov (NCT05078658). The study followed the CONSORT [20] guidelines and was approved by institutional Ethics Committee (EK-667/20). Written informed consent was granted by all the participants and their legal guardians.

### Study protocol

The study protocol is shown in Supplementary Fig. 1. At the screening visit (V-2) and again at the end of each intervention period (V5 and V10), the participants underwent a standard anthropometric evaluation by experienced medical anthropologists. The evaluation included measurements of body height to the nearest 1 mm using wall mounted anthropometer (A-226 manufactured by Trystom, Olomouc, Czechia), body weight in underwear using an electronic scale to the nearest 0.1 kg (TH200, manufactured by Tonava, Upice, Czechia), waist, arm and calf circumference to the nearest 1 mm using tape measure and measurement of skin folds using Harpender caliper. The percentage of body fat was calculated from the skin folds using the method by Slaughter [21], the fat-free arm circumference was estimated using the formula: arm circumference—(skin fold above triceps*3.14). BMI was calculated using the standard equation. In 15 participants that joined the study from January 2023 onward, measurement of body composition was available using bioelectrical impedance InBody 770 (InBody Co., Ltd., Seoul, Rep. of Korea). The body composition was analyzed and numerical values according to age and sex were calculated using integrated InBody software, version 4.0.0.6 (007).

The standard deviation scores (SDS) were calculated for each of the variables using the Czech population data [22]. The participants were further categorized into four BMI SDS categories - normal body weight −1.96 to 1.00 (percentile 5.0–84.0), overweight 1.01–1.50 (percentile 84.1–93.3), obese 1.51–2.00 (percentile 93.4–97.7) and morbidly obese >2.01 (above percentile 97.7) [23].

At the same time points, the participants underwent the evaluation of dynamic muscle function using a jumping mechanography Leonardo Mechanograph Ground Reaction Force Platform (manufactured by Novotec Medical GmbH in Germany). Maximal and relative muscle strength (Fmax, N; resp. Fmax/BW, no unit) and power (Pmax, W; resp. Pmax/mass, W/kg) were evaluated, with the tests used for the evaluation as described elsewhere [24]. Sex- and height-specific standard deviation scores were calculated using our own previously published reference data [25].

### Statistical analysis

The analysis was based on the differences in the outcomes measured at V5 and V10 between the end of the LCD period and the end of the RCD period. The effect of LCD was tested by the Welch two-sample t-test performed on those differences, the confidence interval is based on the Welch t-statistic. The Stuart test was used to evaluate the effect of dietary interventions on the classification of subjects into the BMI categories.

## Results

A total of 34 subjects completed the study. One subject decided to withdraw from the study at V6 (after one week of LCD), the reason being unwillingness to further comply to the dietary restrictions. Twenty subjects were randomized to RCD-first group while fifteen were randomized to LCD-first group. The baseline data for both study groups are shown in Supplementary Table 1. There were no significant differences between both groups in anthropometric data at baseline visit.

**Fig. 1:**
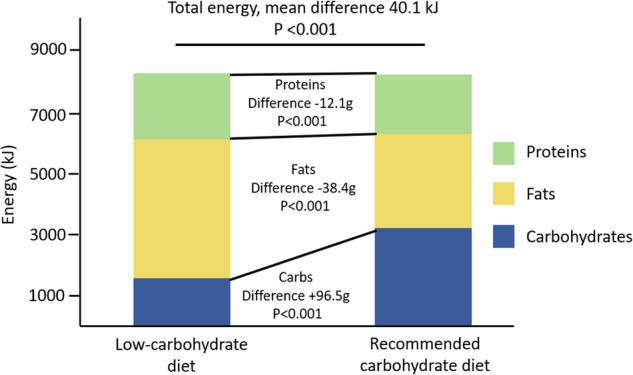
Macronutrient intake during the intervention periods. The amount of energy gained from carbohydrate was decreased during the LCD period while the amount from fat and protein was increased. Although the total energy intake was significantly lower during the LCD period (*P* < 0.001), the difference was not clinically meaningful (–40.1 kJ/day).

There was no clinically meaningful difference in the average daily energy intake between the intervention periods (LCD 8 240 kJ/day, RCD 8 280 kJ/day) despite the statistical significance upon formal testing (*P* < 0.001). The intake of carbohydrates was duly decreased during the LCD period (94.5 g/day vs. 191 g/day while of RCD, *P* < 0.001) while the intake of lipids was higher on LCD (119.0 g/day vs. 80.6 g/day, *P* < 0.001) and so was the intake of proteins (128.3 g/day vs. 116.2 g/day, *P* < 0.001) (Fig. 1).

At the end of the LCD period, the participants had significantly lower body weight and BMI including their respective age and sex specific SD-scores than at the end of the RCD period (61.7 kg vs. 62.6 kg, *P* < 0.001 and 22.3 kg/m^2^ vs. 22.7 kg/m^2^, *P* < 0.001) and (0.84 SD vs. 0.94 SD, *P* < 0.001 and 0.81 SD vs. 0.91 SD, *P* < 0.001). Similarly, the waist (77.6 cm vs. 79.0 cm, *P* = 0.001), arm (28.1 cm vs. 28.6 cm, *P* = 0.003) and calf (36.2 cm vs. 36.5 cm, *P* = 0.004) circumference were significantly lower at the end of the LCD period. This was also true for the age and sex specific SD-scores. The body fat percentage assessed by bioimpedance was significantly lower at the end of the LCD period (24.5% vs. 25.3%, *P* = 0.001) (performed only in 15 subjects). The complete primary results are shown in Table 1 with the data for individual timepoints shown in Supplementary Table 2.

**Table 1:** Anthropometry and body fat percentage bioelectrical impedance measurements at the ends of both intervention periods for each participant.

	End of LCD visit	End of RCD visit	LCD effect (95% CI)	*P* value
Body weight (kg)	61.7 (17.6)	62.6 (17.9)	−0.94 (−1.46 to −0.42)	**<0.001**
Body weight (SDS)	0.84 (1.0)	0.94 (0.99)	−0.11 (−0.15 to −0.06)	**<0.001**
Body height (cm)	164 (12.5)	164 (12.4)	−0.003 (−0.11 to 0.10)	0.954
Body height (SDS)	0.11 (0.63)	0.10 (0.66)	0.015 (−0.03 to 0.05)	0.448
Body mass index (kg/m^2^)	22.3 (4.19)	22.7 (4.26)	−0.36 (−0.53 to −0.19)	**<0.001**
Body mass index (SDS)	0.81 (0.98)	0.91 (0.98)	−0.10 (−0.15 to −0.06)	**<0.001**
Waist circumference (cm)	77.6 (12.7)	79 (12.6)	−1.42 (−2.24 to −0.61)	**0.001**
Waist circumference SDS	0.79 (0.98)	0.94 (0.94)	−0.16 (−0.24 to −0.08)	**0.001**
Arm circumference (cm)	28.1 (4.52)	28.6 (4.57)	−0.49 (−0.79 to −0.18)	**0.003**
Arm circumference SDS	1.34 (1.01)	1.49 (1.00)	−0.15 (−0.25 to −0.05)	**0.004**
Calf circumference (cm)	36.2 (4.47)	36.5 (4.32)	−0.35 (−0.57 to −0.12)	**0.004**
Calf circumference SDS	0.81 (1.01)	0.95 (0.99)	−0.15 (−0.24 to −0.05)	**0.003**
Body fat calculated SDS	0.65 (1.1)	0.74 (1.07)	−0.08 (−0.20 to 0.05)	0.221
Calculated fat−free arm circumference (cm)	23.2 (3.27)	23.4 (3.29)	−0.205 (−0.61 to 0.20)	0.304
Body fat percentage^a^(bioimpedance) (*N* = 15)	24.5 (9.85)	25.3 (9.91)	−0.79 (−1.21 to −0.38)	**0.001**

Body weight, BMI and waist, arm and calf circumference and body fat percentage assessed by bioimpedance were significantly decreased at the end of the LCD period.

Data are shown as means (SD). Statistically significant differences are marked in bold.

^a^For the bioimpedance measurement, only data of the last 15 participants were used.

At the screening visit, 20 (58.8%) participants were in the normal BMI SDS category, 5 (14.7%) in the overweight, 3 (8.8%) were obese and 6 (17.6%) morbidly obese. This distribution was not changed at the end of the RCD period, while at the end of the LCD period, 22 (64.7%) were in the normal, 4 (11.8%) in the overweight, 3 (8.8%) in the obese and 6 (14.7%) in the morbidly obese category. The shifts in the BMI categories were not statistically significant (*P* = 0.392) (Supplementary Table 3).

Key parameters of muscle functions in both intervention groups are detailed in Table 2 with the data for individual timepoints shown in Supplementary Table 4. The analysis showed no differences in maximal (Pmax) or relative (Pmax/mass) power (*P* = 0.487 and *P* = 0.689, respectively) and maximal muscle strength (Fmax) in both legs (*P* = 0.854 and *P* = 0.189, respectively). The only nominally statistically significant difference was found for relative muscle strength (Fmax/BW) in left leg where greater strength was achieved after LCD than RCD (*P* = 0.027).

**Table 2:** Dynamic muscle function data at the ends of both intervention periods.

	End of LCD visit	End of RCD visit	LCD effect (95% CI)	*P* value
Pmax (W)	2.84 (0.99)	2.87 (1.00)	−0.03 (−0.09 to 0.02)	0.208
Pmax SDS	0.56 (1.23)	0.61 (1.32)	−0.06 (−0.24 to 0.12)	0.487
Pmax/mass (W/kg)	44.7 (6.76)	44.4 (6.93)	0.21 (−0.65 to 1.07)	0.617
Pmax/mass SDS	−0.13 (0.82)	−0.17 (0.89)	0.03 (−0.12 to 0.17)	0.689
FmaxL (N)	1.85 (0.51)	1.86 (0.48)	−0.0006 (−0.04 to 0.04)	0.975
FmaxL SDS	0.61 (1.34)	0.59 (1.23)	0.02 (−0.15 to 0.18)	0.854
FmaxL/BW (no unit)	3.04 (0.37)	3.00 (0.35)	0.04 (−0.03 to 0.09)	0.243
FmaxL/BW SDS	−0.29 (1.26)	−0.39 (1.24)	0.10 (−0.09 to 0.30)	0.297
FmaxR (N)	1.87 (0.49)	1.85 (0.47)	0.02 (−0.03 to 0.07)	0.369
FmaxR SDS	0.65 (1.06)	0.54 (1.11)	0.11 (−0.06 to 0.29)	0.189
FmaxR/BW (no unit)	3.06 (0.35)	2.99 (0.35)	0.07 (0.01 to 0.14)	**0.027**
FmaxR/BW SDS	−0.22 (1.16)	−0.44 (1.16)	0.23 (0.03 to 0.43)	**0.027**

Data are shown as means (SD). The standard deviation scores are adjusted for sex and body height. Statistically significant differences are marked in bold.

*BW* body weight, *Fmax* maximal muscle force, *L* left leg, *Pmax* maximal muscle power, *R* right leg, *SDS* standard deviation score.

## Discussion

Our study suggests that a short-term low-carbohydrate diet may decrease body weight and BMI, while not negatively impacting the muscle strength of young persons with T1D. These findings further increase our knowledge about this dietary approach and provides some evidence that may lead toward a more informed decision on the side of the physician as well as the child/caregiver of a child with T1D.

Despite not being recommended by the scientific societies, LCD is considerably popular among people with type 1 diabetes and/or caregivers of children with type 1 diabetes (CwD) [13, 19]. While the improvement in metabolic control is the most commonly cited reason for LCD initiation among the caregivers of CwD, weight and body fat reduction were also often mentioned [13]. This comes as no surprise as the rates of pediatric overweight, obesity and adiposity are increasing in the general population [3] as well as among CwD where the anabolic effects of insulin play an additional role [4]. A lack of approved adjunctive therapy (for example, the glucagon-like peptide receptor agonists) for type 1 diabetes might be one of the reasons driving the increasing popularity of dietary interventions including LCD.

While some of the recent studies focused mostly on metabolic control achieved on LCD [12], [14], less is known about its metabolic effects on anthropometric features, body composition, and muscle strength. There are alarming data that mention stunted growth and severe weight reduction but these were observed exclusively among children on very-low carbohydrate (ketogenic) diets [7, 26]. A less severe carbohydrate reduction might not have such deleterious effects and a moderate reduction in body weight and BMI might be beneficial as seen in adult studies [17, 18, 27]. The decrease in body weight, BMI and body fat percentage in our study was minor but the duration of our intervention was rather short. It is possible that a sustained period of LCD would have an increased impact again suggested by the studies performed in adults [18, 27]. Furthermore, while fatigue and/or weakness were described during LCD [7], there were no studies that would try to objectify these. Our data suggest that these feelings are probably subjective as we have not seen a decrease in muscle strength nor power during the LCD. This is further evidenced by the fact that we have not seen any difference in the quality of life during the intervention periods (data shown elsewhere) [12]. In addition, patients in both LCD and RCD group showed higher relative muscle power when compared to our previous study [28]. This suggests other factors such as glycemic control may play a role in reported fatigue and/or weakness. While BMI was comparable between the studies, HbA1c was significantly lower in the present study (data shown elsewhere).

Our study has several strengths. Firstly, the cross-over design and pre-made ready meal delivery make this study rather robust and eliminate some of the possible biases. Secondly, the very high completion rate suggests that the participants were willing to comply to the nutritional interventions and that they generally saw the weight loss as a benefit and not as a reason to terminate their participation.

There are several limitations to our study. Firstly, the length of the study did not allow us to study whether the observed differences would be sustainable beyond the 5-week period. Yet, the data in adults suggest that this might be the case at least for a period of six months [18]. Also, for the same reason, we were unable to study the effects of LCD on linear growth. Secondly, the intervention compliance was self-reported, but we believe the low initial HbA1c (suggesting excellent compliance to diabetes regimen) and the decrease in the insulin dose throughout the LCD period (data shown elsewhere) [12] allow us to presume high compliance of the study participants. Also, the body composition data were available only in the last 15 participants since we only acquired the bioimpedance device at that time-point. Yet, the results were statistically significant nonetheless, suggesting we might have achieved similar results for the whole cohort. The bioelectrical impedance may be affected by factors such as state of hydration or prior physical activity but we tried to mitigate these differences by measuring the subjects early in the morning in a fasted state. Also, the loss of fat-free mass might have contributed to the decrease in body weight even though we have not seen any statistically significant differences in the calculated fat-free circumference of the arm. Additionally, while the dual X-ray absortptiometry (DXA) measurement is considered the golden standard for body composition estimation, number of studies showed high correlation between DXA and bioelectrical impedance devices, InBody 770 in particular [29–31]. Bioelectrical impedance can be therefore considered a good alternative to DXA in children and adolescents given the lack of radiation exposure. Lastly, despite showing statistically significant differences in the body fat mass measured by bioelectrical impedance, the clinical significance might be minor, especially given the relatively short duration of the interventions.

In conclusion, we demonstrated that a short-term intervention with low-carbohydrate diet is able to decrease body weight, BMI, and body fat percentage in children and young people with T1D without negatively affecting their muscle function.

## Supplementary information

Supplementary material

## Data Availability

All data used for the analysis in this article are available on request from the authors.
