# Supplementary material for: Short-term low-carbohydrate diet decreases body weight and fat mass but not muscle strength in children and young people with type 1 diabetes
Source: Eur J Clin Nutr. 2025 Aug 22;79(11):1149–53. doi: 10.1038/s41430-025-01658-2 (PMC12580311; doi:10.1038/s41430-025-01658-2)
Supplement: Supplementary file 1 — Supplementary material [file 41430_2025_1658_MOESM1_ESM.docx]

**Supplementary Figure 1 – Study protocol**

**
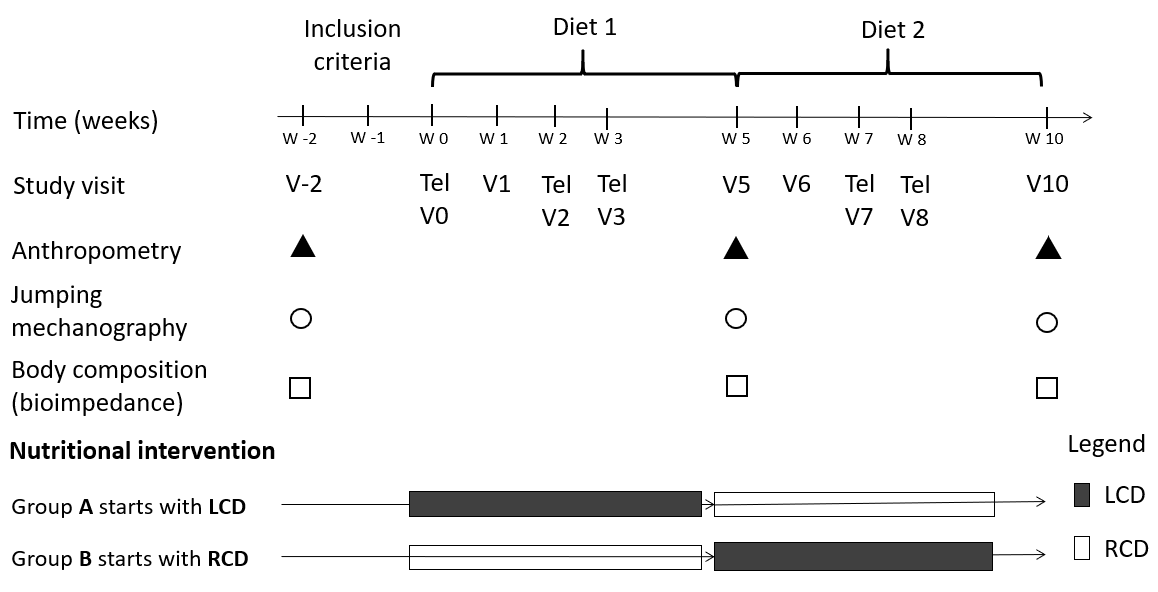
**

**Supplementary Table 1 – Baseline data of the study participants.**

|  | **RCD first** | **LCD first** | ***P*-value** |
| --- | --- | --- | --- |
| **Subjects** | N=20 | N=15 |  |
| **Demographics** |  |  |  |
| Age (years) | 15.0 (2.5) | 14.3 (3.4) | 0.53 |
| Sex | F = 10 (50.0%)  M = 10 (50.0%) | F = 10 (66.7%)  M = 5 (33.3%) | 0.49 |
| T1D duration (years) | 5.8 (3.6) | 4.9 (4.1) | 0.53 |
| **Anthropometric data** |  |  |  |
| Body weight (kg) | 65.0 (17.49) | 59.8 (19.83) | 0.42 |
| Body weight (SDS) | 0.93 (1.15) | 1.07 (0.91) | 0.68 |
| Body height (cm) | 166.8 (10.61) | 159.7 (14.29) | 0.12 |
| Body height (SDS) | 0.08 (0.71) | 0.05 (0.63) | 0.90 |
| Body mass index (kg/m^2^) | 23.0 (4.39) | 22.7 (4.51) | 0.83 |
| Body mass index (SDS) | 0.90 (1.06) | 1.07 (0.85) | 0.61 |
| Tanner stage (F) | B1 – 1  B2 – 0  B3 – 2  B4 – 2  B5 – 5 | B1 – 2  B2 – 1  B3 – 0  B4 – 0  B5 – 7 | NA |
| Tanner stage (M) | G1 – 0  G2 – 2  G3 – 1  G4 – 1  G5 – 6 | G1 – 1  G2 – 1  G3 – 1  G4 – 1  G5 – 1 | NA |
| Waist circumference (cm) | 80.68 (13.92) | 77.23 (13.63) | 0.47 |
| Waist circumference SDS | 0.97 (1.04) | 0.95 (0.99) | 0.96 |
| Arm circumference (cm) | 29.05 (4.32) | 27.79 (5.30) | 0.46 |
| Arm circumference SDS | 1.50 (1.01) | 1.45 (1.12) | 0.91 |
| Calf circumference (cm) | 36.80 (3.79) | 36.00 (5.27) | 0.62 |
| Calf circumference SDS | 0.90 (1.13) | 1.04 (0.95) | 0.68 |
| Calculated fat-free arm circumference (cm) | 23.50 (2.80) | 22.21 (3.76) | 0.27 |
| Body fat calculated SDS | 1.00 (1.09) | 1.11 (1.31) | 0.79 |
| Body fat percentage (bioimpedance)  RCD first (N=8), LCD first (N=7) | 29.71 (11.61) | 21.36 (4.49) | 0.09 |
| **Dynamic muscle function data** |  |  |  |
| Pmax (W) | 2.93 (0.96) | 2.70 (1.09) | 0.53 |
| *Pmax SDS* |  |  |  |
| Pmax/mass (W/kg) | 43.2 (7.19) | 43.8 (5.71) | 0.80 |
| *Pmax/mass SDS* |  |  |  |
| FmaxL (N) | 1.91 (0.44) | 1.87 (0.67) | 0.84 |
| *FmaxL SDS* |  |  |  |
| FmaxL/BW (no unit) | 2.93 (0.36) | 3.17 (0.26) | **0.03** |
| *FmaxL/BW SDS* |  |  |  |
| FmaxR (N) | 1.99 (0.54) | 1.83 (0.69) | 0.49 |
| *FmaxR SDS* |  |  |  |
| FmaxR/BW (no unit) | 2.97 (0.39) | 3.12 (0.33) | 0.21 |
| *FmaxR/BW SDS* |  |  |  |
| **Treatment-related data** |  |  |  |
| Treatment modality | MDI = 6 (30%)  CSII = 14 (70%) | MDI = 8 (53%)  CSII = 7 (47%) | 0.16 |
| Daily insulin dose (IU/kg/day) | 0.92 (0.35) | 0.69 (0.27) | **0.04** |
| HbA1c (mmol/mol) | 48.2 (6.91) | 49.8 (12.07) | 0.65 |
| HbA1c (%) | 6.6 (2.78) | 6.7 (3.25) | 0.65 |

Data are shown as means (SD). The standard deviation scores in dynamic muscle function data are adjusted for sex and body height. Statistically significant differences are marked in bold.

BW = body weight, Fmax = maximal muscle force, L = left, Pmax = maximal muscle power, R = right, SDS = standard deviation score

**Supplementary Table 2 – Anthropometry and body fat percentage bioelectrical impedance measurements throughout the course of the study.**

| Variable | Means baseline and 5 weeks LCD | Difference baseline and 5 weeks LCD | Means baseline and 5 weeks RCD | Difference baseline and 5 weeks RCD | Means  week 5 and week 10 LCD | Difference week 5 and week 10 LCD | Means  week 5 and week 10 RCD | Difference week 5 and week 10 RCD |
| --- | --- | --- | --- | --- | --- | --- | --- | --- |
| Body height (cm) | 160 160 | 0.53 | 167 167 | 0.55 | 167 168 | 0.25 | 160 160 | 0.25 |
| Body height (SDS) | 0.0467 0.1 | 0.053 | 0.075 0.09 | 0.015 | 0.09 0.116 | -0.011 | 0.1 0.06 | -0.04 |
| Body weight (kg) | 59.8 59 | -0.73 | 65 64.4 | -0.69 | 64.4 63.7 | -0.88 | 59 60 | 1 |
| Body weight (SDS) | 1.07 0.94 | -0.13 | 0.93 0.84 | -0.09 | 0.84 0.753 | -0.12 | 0.94 1.04 | 0.1 |
| Body mass index (kg/m^2^) | 22.7 22.3 | -0.41 | 23 22.7 | -0.35 | 22.7 22.3 | -0.37 | 22.3 22.6 | 0.35 |
| Body mass index (SDS) | 1.07 0.94 | -0.13 | 0.905 0.795 | -0.11 | 0.795 0.705 | -0.095 | 0.94 1.05 | 0.11 |
| Waist circumference (cm) | 77.2 75.7 | -1.5 | 80.7 79.7 | -0.71 | 79.7 79.2 | -1.1 | 75.7 77.5 | 1.8 |
| Waist circumference SDS | 0.953 0.793 | -0.16 | 0.97 0.837 | -0.089 | 0.837 0.8 | -0.11 | 0.793 1 | 0.21 |
| Arm circumference (cm) | 27.8 27.5 | -0.34 | 29.1 29 | -0.08 | 29 28.7 | -0.28 | 27.5 28.1 | 0.69 |
| Arm circumference SDS | 1.45 1.34 | -0.11 | 1.5 1.44 | -0.05 | 1.44 1.34 | -0.084 | 1.34 1.56 | 0.22 |
| Calf circumference (cm) | 36 35.7 | -0.26 | 36.8 36.7 | -0.06 | 36.7 36.5 | -0.21 | 35.7 36.2 | 0.49 |
| Calf circumference SDS | 1.04 0.893 | -0.15 | 0.895 0.815 | -0.08 | 0.815 0.742 | -0.11 | 0.893 1.08 | 0.19 |
| Body fat calculated SDS | 22.2 22.6 | 0.37 | 23.5 23.5 | -0.014 | 23.5 23.7 | 0.15 | 22.6 23.1 | 0.56 |
| Calculated fat-free arm circumference (cm) | 1.11 0.696 | -0.41 | 0.996 0.816 | -0.18 | 0.816 0.621 | -0.14 | 0.696 0.713 | 0.017 |
| Body fat percentage †(bioimpedance) (N=15) | 21.4 20.6 | -0.74 | 29.7 29 | -0.69 | 29 27.8 | -1.2 | 20.6 21 | 0.4 |

Data are shown as means

† For the bioimpedance measurement, only data of the last 15 participants were used.

**Supplementary Table 3 – Distribution of BMI SDS categories at the screening visit and at the ends of the intervention periods.**

There was no significant difference in the category distribution between the diets (*P*=0.392).

| **BMI SDS**  **category (N) (%)** | **Screening** | **End of LCD period** | **End of RCD period** |
| --- | --- | --- | --- |
| **Normal**  **(-1.96-1.00 SDS)** | 20 (58.8%) | 22 (64.7%) | 20 (58.8%) |
| **Overweight**  **(1.01-1.50 SDS)** | 5 (14.7%) | 4 (11.8%) | 5 (14.7%) |
| **Obese**  **(1.51 – 2.00 SDS)** | 3 (8.8%) | 3 (8.8%) | 3 (8.8%) |
| **Morbidly obese (>2.01 SDS)** | 6 (17.6%) | 5 (14.7%) | 6 (17.6%) |

**Supplementary Table 4 – Dynamic muscle function data throughout the course of the study.**

| Variable | Means baseline and 5 weeks LCD | Difference baseline and 5 weeks LCD | Means baseline and 5 weeks RCD | Difference baseline and 5 weeks RCD | Means  week 5 and week 10 LCD | Difference week 5 and week 10 LCD | Means  week 5 and week 10 RCD | Difference week 5 and week 10 RCD |
| --- | --- | --- | --- | --- | --- | --- | --- | --- |
| Pmax (W) | 2.7 2.72 | 0.025 | 2.93 2.92 | -0.014 | 2.92 2.84 | 0.0039 | 2.72 2.77 | 0.071 |
| *Pmax SDS* | 1.1 1.05 | -0.05 | 0.347 0.179 | -0.17 | 0.179 0.147 | 0.0056 | 1.05 1.11 | 0.13 |
| Pmax/mass (W/kg) | 43.8 45 | 1.2 | 43.2 43.5 | 0.27 | 43.5 44 | 0.94 | 45 45.1 | 0.51 |
| *Pmax/mass SDS* | 0.05 0.229 | 0.18 | -0.495 -0.516 | -0.021 | -0.516 -0.416 | 0.13 | 0.229 0.227 | 0.071 |
| FmaxL (N) | 1.87 1.8 | -0.074 | 1.91 1.88 | -0.029 | 1.88 1.9 | 0.0028 | 1.8 1.8 | 0.004 |
| *FmaxL SDS* | 1.32 0.98 | -0.34 | 0.426 0.274 | -0.15 | 0.274 0.294 | -0.017 | 0.98 0.933 | -0.047 |
| FmaxL/BW (no unit) | 3.17 3.09 | -0.08 | 2.93 2.93 | -0.00053 | 2.93 2.99 | 0.047 | 3.09 3.07 | -0.023 |
| *FmaxL/BW SDS* | 0.233 -0.007 | -0.24 | -0.695 -0.679 | 0.016 | -0.679 -0.528 | 0.11 | -0,107 | -0.093 |
| FmaxR (N) | 1.83 1.8 | -0.024 | 1.99 1.88 | -0.1 | 1.88 1.86 | 0.017 | 1.8 1.78 | -0.024 |
| *FmaxR SDS* | 1.24 1.03 | -0.21 | 0.637 0.247 | -0.39 | 0.247 0.332 | 0.078 | 1.03 0.879 | -0.15 |
| FmaxR/BW (no unit) | 3.12 3.14 | 0.015 | 2.97 2.93 | -0.036 | 2.93 3 | 0.061 | 3.14 3.05 | -0.084 |
| *FmaxR/BW SDS* | 0.1 0.0929 | -0.0071 | -0.563 -0.689 | -0.13 | -0.689 -0.453 | 0.21 | 0.0929 -0.157 | -0.25 |

Data are shown as means. BW = body weight, Fmax = maximal muscle force, L = left leg, Pmax = maximal muscle power, R = right leg, SDS = standard deviation score
